# Fulminant Myocarditis and Venoarterial Extracorporeal Membrane Oxygenation: A Systematic Review

**DOI:** 10.7759/cureus.54711

**Published:** 2024-02-22

**Authors:** Spencer E Briglio, Viraj Khanduja, Justin D Lothan, Vasavi Rakesh Gorantla

**Affiliations:** 1 Anatomical Sciences, St. George's University School of Medicine, True Blue, GRD; 2 Anatomy, St. George's University, True Blue, GRD; 3 Anatomical Sciences, St. George's University, True Blue, GRD

**Keywords:** venoarterial extracorporeal membrane oxygenation, mechanical circulatory assistance, left ventricular ejection fraction, cardiogenic shock, fulminant myocarditis

## Abstract

This systematic review aimed to look at the effectiveness of venoarterial extracorporeal membrane oxygenation (VA-ECMO) therapy in treating fulminant myocarditis and evaluating the optimal length of time a patient should be placed on VA-ECMO. Fulminant myocarditis is a potentially life-threatening medical condition most commonly brought on by cardiogenic shock, which often progresses to severe circulatory compromise, requiring the patient to be placed on some form of mechanical circulatory assistance to maintain adequate tissue perfusion. Medical centers have multiple mechanical assistive devices available for treatment at their disposal, but our area of focus was placed on one system in particular: VA-ECMO therapy. Although the technology has been around for more than 30 years, there is limited information on how effective VA-ECMO is regarding the treatment of fulminant myocarditis. Due to the lack of data regarding the treatment administration of VA-ECMO for fulminant myocarditis, standard treatment duration guidelines do not exist, resulting in a wide variation of treatment administrations among medical centers. In regard to short-term outcomes, VA-ECMO has shown to be effective in treating fulminant myocarditis, with a one-year post-hospital survival rate ranging from 57.1% to 78% at discharge. For long-term health and survival, the studies that recorded long-term survival ranged from 65% to 94.1%. However, given the small number of studies that pursue this, more research is needed to prove the efficacy of VA-ECMO for the treatment of fulminant myocarditis.

## Introduction and background

Fulminant myocarditis is a specific subset of myocarditis characterized by a compromise of cardiac function due to rapidly deteriorating inflammation, which often progresses to critical health emergencies in patients, such as cardiogenic shock and cardiac arrest [1]. The initial inflammatory response is due to the marked elevation of cytokines, leading to the direct cytotoxicity from pathogens responsible for the symptoms of fulminant myocarditis [1]. Fulminant myocarditis can be identified as an acute illness lasting at least two weeks that has caused hemodynamic instability, such as an arrhythmia or cardiogenic shock, requiring the patient to be given hemodynamic support, such as a ventricular assistive device (VAD) or inotropes [2]. 

Diagnosis criteria are characterized by a decreased left ventricular ejection fraction (<50%), along with the patient presenting with cardiogenic shock due to infection and needing a form of mechanical circulatory support in order to maintain central and peripheral blood flow [1]. This mechanical support is necessary to maintain total patient tissue perfusion in fulminant myocarditis-induced cardiac arrest or cardiac shock while the patient is bridged to recovery via medical interventions aimed at alleviating the fulminant myocarditis. One of the predominant forms of mechanical circulatory support is venoarterial extracorporeal membrane oxygenation (VA-ECMO), which removes carbon dioxide using an oxygenator and oxygenates the patient’s venous blood before returning it via arterial circulation [3]. VA-ECMO seeks to decrease the hospital stay of the patient, increase their left ventricular ejection fraction, and eventually lead to recovery without the need for a VAD or heart transplant [2]. Due to the heightened cost and risk factors of using VA-ECMO, such as the increased risk of vascular, neurological, or infectious complications, it is pivotal that VA-ECMO is administered by a well-trained team paired with a particular patient selection that has favorable outcomes [4]. 

Fulminant myocarditis is typically viewed as a lethal disease, but with appropriate therapeutic treatments, such as mechanical cardiopulmonary support, patients can often be bridged to a full recovery [2,4]. Clinical guidelines for the administration of VA-ECMO for the treatment of patients with fulminant myocarditis have not yet been established, particularly in regard to treatment duration. In order to optimize clinical prognosis and minimize post-treatment complications, analyzing the long-term follow-up of patients who have been treated with VA-ECMO can give us a greater insight into the ideal duration of administering this treatment to give the patient the highest chance of survival. The aim of this systematic review is to look at both short-term treatment and long-term outlook of patients who were treated with VA-ECMO for fulminant myocarditis to determine whether VA-ECMO is effective and whether any clinical guidelines regarding treatment duration along with long-term health outcomes can be implemented to more effectively treat and manage symptoms on patients with fulminant myocarditis. 

**Figure 1:**
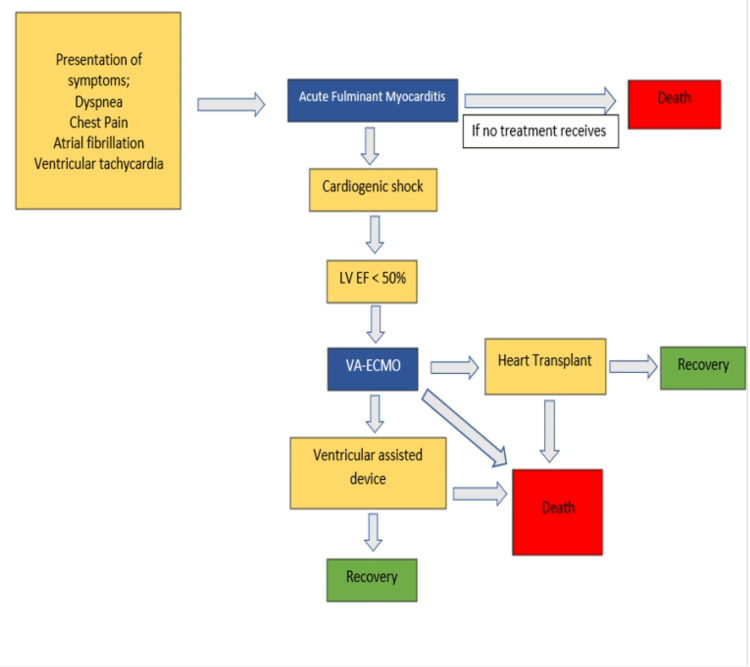
Flowchart indicating the symptoms, diagnostic criteria, and treatments of fulminant myocarditis This chart describes the different potential steps in describing and diagnosing fulminant myocarditis and the possible outcomes that can arise from fulminant myocarditis [1]. VA-ECMO, venoarterial extracorporeal membrane oxygenation; LVEF, left ventricular ejection fraction

**Figure 2:**
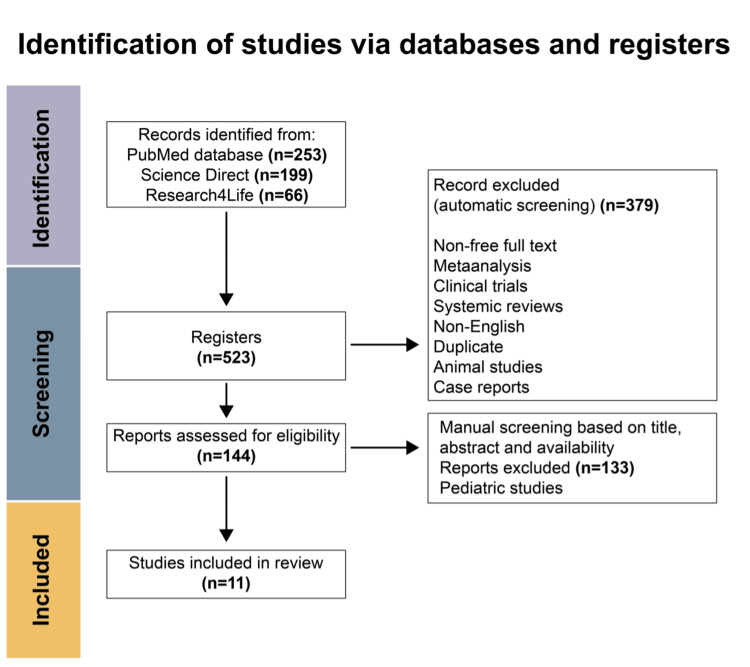
PRISMA flowchart describing the literature screening of studies concerning the use of VA-ECMO in patients with fulminant myocarditis Screening of the literature has been done, mirroring the protocol described in the PRISMA statement [5]. Original Article: https://doi.org/10.1371/journal.pmed.1000100 VA-ECMO, venoarterial extracorporeal membrane oxygenation

This systematic review follows the Preferred Reporting Items for Systematic Reviews and Meta-Analyses (PRISMA) guidelines [5]. A literature search was conducted on October 5, 2022, through the following databases: Medline PubMed, Research4Life, and ScienceDirect. The following inquiry was conducted on all three online databases with these keywords: “Fulminant Myocarditis” AND “Veno arterial extracorporeal membrane oxygenation” AND “VA-ECMO” AND “VA ECMO.” Even after an extensive and comprehensive search, we acknowledge that not every relevant research study may have been incorporated into this systematic review. A total of 523 publications were found and screened. The inclusion and exclusion criteria were applied to these databases, and only the articles relevant to our topic of study were considered. A total of 11 publications were used (Figure 1). Figure 2 depicts the progression of fulminant myocarditis and the possible outcomes that would arise, along with the main presentation of symptoms of fulminant myocarditis. 

Inclusion criteria 

The articles included in our study were publications written in English, articles that provided free-full text, clinical trials, research articles, custom dates (1992-2022), and scholarly and peer-reviewed articles. 

Exclusion criteria 

All non-English publications, animal studies, case reports, meta-analyses, and duplicate articles between the three different search engines were excluded from our study. Studies done on pediatrics were excluded as well due to the topic of research focusing solely on the adult population. Articles that discussed or utilized other treatments from VA-ECMO were also excluded from our study. Other cardiopulmonary diseases that used VA-ECMO were excluded from our study as well. A summary of the exclusion criteria is summed up in Figure 1 [5]. 

Bias 

After all the studies were chosen from the three different databases, the three co-authors graded the risk bias of each individual study picked using the Grading of Recommendations Assessment, Development, and Evaluation (GRADE) system. The GRADE system was utilized in order to analyze any study flaws that included risk and publication bias as well as to help eliminate any articles that did not fit the criteria of this study. Two of the co-authors would have applied the inclusion and exclusion criteria to an article, and if any inconsistency presented itself, the third co-author would provide the final outcome after the assessment of the study. 

## Review

Results

After thoroughly searching the literature, our initial search yielded 523 articles: 258 articles from Medline PubMed, 199 articles from ScienceDirect, and 66 articles from Research4Life. Upon initial screening, 379 articles were excluded based off the exclusion criteria (non-free full text, meta-analysis, clinical trials, systematic reviews, non-English text, duplicate articles, animal studies, and case reports). This yielded 144 studies to be checked for eligibility. After conducting a manual screening based on title, abstract, and availability, we were left with 11 studies that were to be included in this review. Of these 11 articles that were included, nine were retrospective studies and two were comparative studies. The characteristics of each study are shown in Table 1.

**Table 1:** Fulminant myocarditis outcomes The table describes survival rates and various treatment outcomes in patients diagnosed with fulminant myocarditis who were treated with VA-ECMO VA-ECMO, venoarterial extracorporeal membrane oxygenation

Author	N and type of study	Origin	Treatment/management	Findings	Follow-up information, outcome, or prognosis
Asaumi et al., 2005 [7]	14, comparative study (1993-2001)	Japan	VA-ECMO	After a mean VA-ECMO treatment time of 130 hours, 71% of patients survived to discharge. Upon a mean follow-up of 66 months, 1 patient died of heart failure.	Effective treatment: 71% lived to discharge, with only 1 death reported 15 months later.
Mirabel et al., 2011 [8]	35, comparative study (2003-2009)	France	VA-ECMO	Over a 6-year period, 35 patients were admitted to the ICU and put on VA-ECMO, with a 68.6% survival rate at 22 months post-discharge (24 survived, 11 died).	Treatment was effective, with a mean VA-ECMO usage of 16 days in the survival and non-survival groups.
Lo Coco et al., 2018 [10]	57, retrospective multicenter center (2008-2013)	Italy	VA-ECMO	After mean VA-ECMO treatment of 9.9 days, survival occurred in 71.9%, and upon median 15-month follow-up, 2 deaths were reported. 5-year survival for treated patients at 65.2%.	Effective treatment: weaned off 75% of patients with only 2 late deaths.
Chong et al., 2018 [15]	35, retrospective single-center study (2003-2017)	Taiwan	VA-ECMO	Over a 14-year period, 35 patients with acute fulminant myocarditis were put on VA-ECMO for an average of 6.5-9.5 days and showcased a 57.1% in-hospital survival rate (20 survived at post 1-year checkup, 15 died). The age, race, gender, and preexisting comorbidities between the two groups were similar.	VA-ECMO is an effective treatment because the majority of the group survived with treatment; however, the severity of the preexisting comorbidities is still a major factor in survival.
Aoyama et al., 2002 [9]	52, guideline (special report) (1997-2000)	Japan	VA-ECMO	Out of 52 patients, 30 survived, 21 died, and 1 remained in a vegetative state. 30 surviving patients had 10% mortality over the average 962-day follow-up period.	Does not mention the length of treatment.
Gariboldi et al., 2010 [14]	10, retrospective study (2006-2008)	France	VA-ECMO	Over a two-year period, 10 patients were placed on VA-ECMO with a 75% in-hospital survival rate (9 survived, 3 died). The study also showed a 50% success rate of weaning the patients off VA-ECMO after a mean of 12 days.	The in-hospital survival rate showed VA-ECMO is successful in the short term, but no long-term follow-up was done on these patients.
Lee et al., 2021 [11]	71, retrospective single-center cohort study (2004-2019)	South Korea	VA-ECMO	Over the course of 15 years, there was a 71.2% survival rate of the patients who were on VA-ECMO (51 survived, 20 died). A follow-up was done of the VA ECMO group with a mean average time of 456 days, with 3 of the 51 survivors at discharge died.	With a 71.2% in-hospital survival rate and a 94.1% survival rate at 1.5 years post-discharge, VA-ECMO was an effective treatment for fulminant myocarditis.
Ho et al., 2022 [16]	28, retrospective single-center cohort study 2013-2019	Taiwan hospital	VA-ECMO	After treatment with VA-ECMO, 78% of patients survived with a mean time of treatment for survivors of 4.5 days; included in survivors are 2 patients and 5 bridged to heart transplant.	Treatment effective: 78% survival rate, and no follow-up was conducted.
Chou et al., 2020 [13]	88, retrospective analysis (2006-2018)	Taiwan	VA-ECMO	Survival rate of 57% was observed among 88 patients receiving VA-ECMO treatment for a mean of 7 days in surviving patients.	Treatment less effective and slightly longer than seen in other studies.
Montero et al., 2018 [17]	13* (11 VA-ECMO), retrospective multicenter study	France	VA-ECMO	Out of 11 patients with giant-cell fulminant myocarditis, 8 patients received heart transplant (1 patient received total artificial heart), and they were discharged and alive (72.72%) (42 months of mean follow-up time-period)	Patients with a rare form of giant-cell fulminant myocarditis were treated with VA-ECMO and bridged to heart transplant, and it has a survival rate of 72.72%.
Nakamura et al., 2015 [12]	22, retrospective single-center cohort study (1999-2013)	Japan	VA-ECMO	Over 15 years, 22 patients with FM were treated with VA-ECMO, yielding a 59% survival rate (13 survived, 9 died). Additionally, the mean age of the survivors was 36.5, whereas the mean age of those who died was 60.2 years, with both groups being on VA-ECMO for similar times (181 ± 22 and 177 ± 31 hours)	With a survival rate of 59%, VA-ECMO is effective; however, age plays a major factor in treatment, with the younger population having a greater chance and being receptive to the treatment.

Discussion

VA-ECMO is often regarded as the first option life support tool as total hemodynamic support is provided in addition to oxygen and carbon dioxide gaseous exchange for tissue perfusion [6]. Many studies that were reviewed in this article showcase the effectiveness of VA-ECMO treatment short term by in-patient survival rate, along with demonstrating significantly better prognoses of those who have fulminant myocarditis being treated with VA-ECMO than those who have the same condition without receiving VA-ECMO [7]. A study done by Asaumi et al. showed a 71% survival rate that was achieved in patients diagnosed with fulminant myocarditis and subsequently treated with VA-ECMO [7]. Similarly, Mirabel et al. found a 68% ICU survival rate of those who underwent treatment with VA-ECMO, with 10% of that group undergoing a heart transplant [8]. Although these studies mention patient discharge and short-term follow-up in Table 1, nothing is mentioned about whether they can return to their daily lives and routines. Aoyama et al. found a 57.7% survival rate (in-patient short term) of patients who were treated for fulminant myocarditis with VA-ECMO and were able to return to their daily lives [9]. 

Although VA-ECMO remains one of the most effective treatment options available for fulminant myocarditis, the treatment is invasive, and long-term prognosis (more than one year) and complications remain largely unstudied. In a study and follow-up of patients with fulminant myocarditis treated with VA-ECMO, a survival rate of approximately 70% was observed. Upon a mean follow-up of two years, two additional patients died, bringing the long-term survival of VA-ECMO-treated patients to approximately 65% [10]. In a study of 71 patients diagnosed with fulminant myocarditis treated with VA-ECMO, 51 patients survived to discharge [11]. Upon a mean follow-up period of 456 days, three additional patients died, bringing the total survival rate of VA-ECMO treatment at the hospital and post-discharge period of approximately 456 days to approximately 71% [11]. Additionally, Mirabel et al. recorded a follow-up at a mean of 525 days, and 93% of the patients that were discharged were available for follow-up [8]. In the study done by Aoyama et al., these patients were assessed long term, with a mean of 962 days [9]. During this length of time, there was a 10% mortality rate, with three of the 30 patients who survived being readmitted to the ICU (two for congestive heart failure and one for exacerbation of myocarditis) [9]. Asaumi et al. also indicated that one of the 14 patients developed congestive heart failure after discharge, with no indication that it is due to VA-ECMO, something that may need to be explored further in the long-term [7]. Another major complication of VA-ECMO includes leg ischemia, bleeding, stroke, and multiple organ failure [12]. 

The other major factor that we need to look at is the length of treatment and how long a patient with fulminant myocarditis should be treated with VA-ECMO. 

The optimal treatment length of VA-ECMO administered to patients with fulminant myocarditis remains unclear. In a retrospective study of 88 patients with fulminant myocarditis who received VA-ECMO, 49 survived to discharge [13]. The duration of VA-ECMO administered to these patients was an average of seven days. This treatment duration is longer than treatment durations commonly seen in VA-ECMO administration for fulminant myocarditis [13]. The treatment duration for non-surviving patients treated with VA-ECMO was slightly longer at approximately 11.7 days [13]. However, in a study done by Gariboldi et al., the in-patient survival rate of fulminant myocarditis treated with VA-ECMO was 75% (nine of 12), with the average duration of those patients being on the mechanical circulatory support being 12 days [14]. Another study that measured the length of time for VA-ECMO was done by Chong et al. and found that patients with AFM that shared common demographics, such as age, race, gender, and preexisting comorbidities, had a 57.1% (20 of 35 patients) survival rate [15]. Upon a one-year follow-up, those that had survived in-patient and at the one-year mark were placed on VA-ECMO in-patient for an average duration of 6.5-9.5 days [15]. This leaves a lot to be investigated in terms of the duration of treatment and whether patients need to be placed on a type of ventricular device or undergo a heart transplant post-VA-ECMO. 

In a study where 28 patients were treated with VA-ECMO for AFM, six patients died during initial VA-ECMO support. Among 22 survivors, 15 patients recovered completely and were weaned from VA-ECMO. Two patients received VAD, and five patients underwent cardiac transplantation surgery [16]. In all, 78.5% was the survival rate for that cohort. It is worth noting that the percentage of patients who received steroids or IVIG, LVEF and laboratory data at baseline, and the incidence of GI bleeding were similar in both survival and non-survival groups [16]. 

Age and health play a role in the survival of fulminant myocarditis with VA-ECMO, as shown by Nakamura et al. [12]. The younger the patient is, the better the prognosis [12]. In a study by Nakamura et al., there were 22 patients with fulminant myocarditis treated with VA-ECMO, with a survival rate of 59% (13 patients survived and nine non-survival patients) [12]. The mean age of survivors was 36.5, whereas the mean age of non-survivors was 60.2 years, with the duration of treatment with VA-ECMO being similar (181 ± 22 for the survival group and 177 ± 31 hours for the non-survival group) [12]. 

Giant-cell myocarditis (GCM) is a type of fulminant myocarditis characterized by a predominance in affecting young and healthy people. Although it is often fatal, GCM can be treated by administering VA-ECMO and treating underlying causes, giving the patient time to recover in the absence of significant cardiac function [17]. Eleven patients diagnosed with GCM were treated with VA-ECMO, and eight were successfully bridged to transplant, demonstrating a positive prognosis utilizing VA-ECMO in an otherwise fatal disease [17]. 

Patients who did not survive VA-ECMO treatment for fulminant myocarditis showed greater initial and 24-hour serum lactic acid levels, greater cardiac biomarkers, and low pH values following ECMO [15]. The occurrence of acute kidney injury and following hemodialysis were also higher in this group [15]. Levels of CK-MB, troponin I, BUN, and creatinine were highly increased in non-survival patients [16]. 

VA-ECMO is one of the newer and more effective treatments for patients who present with fulminant myocarditis. The implementation of VA-ECMO for patients with fulminant myocarditis provided a majority inpatient survival rate and a decreased need for another type of VAD to help promote blood flow and proper oxygenation [16,15]. Additionally, looking at the long-term outcomes of FM patients who were treated with VA-ECMO was pivotal in seeing if this could be the gold standard for future incidences [11]. With a >70% long-term prognosis, this can encourage other institutions, hospitals, and health care centers to consider and justify VA-ECMO as a viable treatment option [11]. Table 1 summarizes all the different studies done with regards to in-patient, short-term, and long-term survival rate. The length of the treatment with VA-ECMO differs in different studies, with the shortest mean duration being 4.5 days [16] and the longest being 16 days [8]. So, the length of the treatment with VA-ECMO mainly depends upon the severity of patients’ presentation and the underlying comorbidities.

Limitations

As this is a review article, the data used is obtained from a variety of studies. The various medical centers and hospitals where data is obtained may have differing medical procedures and diagnosis standards in regard to fulminant myocarditis. For example, some hospitals may confirm a diagnosis of fulminant myocarditis with a histological test, while others may diagnose solely based on patient presentation and symptoms. In regard to administering VA-ECMO, several limitations exist, such as the cost of treatment, the lack of information pertaining to optimal treatment length, and post-treatment safety complications that the patient may encounter. Due to the high cost of the VA-ECMO pumps and the significant time commitment a VA-ECMO treatment may entail, the financial strain may be a drawback for the medical institution and the patient when considering treatment via VA-ECMO. Due to the rare nature of fulminant myocarditis, data on optimal treatment protocol and long-term patient safety remains sparse. Varying treatment duration standards among hospitals and medical institutions is a drawback because each institution uses VA-ECMO based on symptoms rather than a certain time length protocol, which makes creating a protocol for the length of duration difficult to create.

## Conclusions

In conclusion, there is a sufficient amount of evidence that shows that the treatment of fulminant myocarditis with VA-ECMO is an effective short-term treatment (less than one year), with a short-term survival rate ranging from 57% to 78%. Besides the possible long-term complications, it is important- to note the high long-term survival rate (more than one year), which serves as an indicator that VA-ECMO can be effective for treating fulminant myocarditis. The existing information on the patient’s long-term survival rate is promising and does show a positive long-term prognosis (more than one year); however, more exploration is necessary for the long-term efficacy of VA-ECMO as a treatment for fulminant myocarditis. With regards to treatment duration, we cannot provide a guideline or proper length of time a patient should be placed on fulminant myocarditis due to the sparse data that is available, as well as the existing information not providing any concrete data. Moving forward, harnessing more data for long-term health (survival rate) along with increased documentation of treatment duration will be a strong indicator of how effective VA-ECMO is in relation to fulminant myocarditis.
